# Synthesis and Characterization of the Ligand Based on Benzimidazole and Its Copper Complex: DNA Binding and Antioxidant Activity

**DOI:** 10.1155/2011/105431

**Published:** 2011-11-21

**Authors:** Huilu Wu, Fan Kou, Fei Jia, Bin Liu, Jingkun Yuan, Ying Bai

**Affiliations:** School of Chemical and Biological Engineering, Lanzhou Jiaotong University, Lanzhou 730070, China

## Abstract

A new copper(II) complex with formulae of [Cu(buobb)_2_](pic)_2_, where buobb stands for the ligand of 1,3-bis(1- butylbenzimidazol-2-yl)-2-oxopropane and pic represents 2,4,6-trinitrophenol, has been synthesized and characterized by elemental analyses, molar conductivity, IR, UV-Vis spectra measurements, and cyclic voltammetry. The crystal structure of the copper(II) complex has been determined by X-ray single-crystal diffraction. The coordination environment around each copper(II) atom can be described as a distorted octahedral geometry. The *π*-*π* stacking interactions link the copper(II) complex into a 1D infinite network. The interactions of the ligand and the copper(II) complex with calf thymus DNA (CT-DNA) are investigated by using electronic absorption titration, ethidium bromide-DNA displacement experiments, and viscosity measurements. Additionally, the copper(II) complex's antioxidant properties have been investigated in vitro.

## 1. Introduction

Recently, a lot of beautiful complexes of ingenious design based on flexible bis(imidazole) and bis(triazole) ligands have been crystallographically characterized by different groups [1, 2]. However, bis(benzimidazole) ligands, which represent a class of aromatic N-donor organic linkers, are still less developed [3, 4]. Benzimidazole and its derivative are an important class of aromatic heterocyclic compounds with broad spectrum of biological activities such as antimicrobial [5], anticancer [6], anti-inflammatory [7], antivirus [8], anticonvulsant [9]. DNA is an important cellular receptor; and many chemicals exert their antitumor effects by binding to DNA there by changing the replication of DNA and inhibiting the growth of the tumor cells, which is the basis of designing new and more efficient antitumor drugs and their effectiveness depends on the mode and affinity of the binding [10, 11]. During the past decades, identifying small molecules that are capable of binding to DNA through an intercalation mode has attracted considerable interests [12]. Studies on the interaction of transition metal complexes with nucleic acid have gained prominence, because of their relevance in the development of new reagents for biotechnology and medicine [13]. 

In previous papers, we investigated to a series of V-shaped bis-benzimidazole ligands and their complex [14–17]. In this study, a new ligand and its Cu(II) complex have been synthesized and characterized. The DNA-binding behaviors and antioxidation were investigated.

## 2. Experimental

### 2.1. Materials-Instrumentation-Physical Measurements

The C, H, and N elemental analyses were determined using a Carlo Erba 1106 elemental analyzer. Electrolytic conductance measurements were made with a DDS-11A type conductivity bridge using 10^−3 ^mol*·*L^−1^ solutions in DMF at room temperature. The IR spectra were recorded in the 4000–400 cm^−1^ region with a Nicolet FT-VERTEX 70 spectrometer using KBr pellets. A glassy carbon working electrode, a platinum wire auxiliary electrode, and a saturated calomel electrode (SCE) reference electrode were used in the three*­* electrode measurements. Electronic spectra were taken on a Lab-Tech UV Bluestar spectrophotometer. ^1^H NMR spectra were obtained with a Mercury plus 400 MHz NMR spectrometer with TMS as internal standard and DMSO-d_6_ as solvent. The fluorescence spectra were recorded on a LS-45 spectrofluorophotometer. The antioxidant activities containing the hydroxyl radical (OH^•^) and superoxide anion radical (O_2_^−•^) were performed in water bath with 722 sp spectrophotometer.

Calf thymus DNA (CT-DNA), ethidium bromide (EB), nitroblue tetrazolium nitrate (NBT), methionine (MET), and riboflavin (VitB_2_) were obtained from Sigma-Aldrich Co. (USA). EDTA and safranin were produced in China. Other reagents and solvents were reagent grade obtained from commercial sources and used without further purification. Tris-HCl buffer, Na_2_HPO_4_–NaH_2_PO_4_ buffer, and EDTA-Fe(II) solution were prepared using bidistilled water. The stock solution of complex was dissolved in DMF at 3 × 10^−3^ mol*·*L^−1^. All chemicals used were of analytical grade. The experiments involving interaction of the ligand and the complex with CT-DNA were carried out in doubly distilled water buffer containing 5 mM Tris and 50 mM NaCl and adjusted to pH 7.2 with hydrochloric acid. A solution of CT-DNA gave a ratio of UV absorbance at 260 and 280 nm of about 1.8-1.9, indicating that the CT-DNA was sufficiently free of protein [18]. The CT-DNA concentration per nucleotide was determined spectrophotometrically by employing an extinction coefficient of 6600 M^−1 ^cm^−1^ at 260 nm [19]. 

#### 2.1.1. DNA-Binding Studies

Absorption titration experiments were performed with fixed concentrations of the compounds, while gradually increasing the concentration of CT-DNA. To obtain the absorption spectra, the required amount of CT-DNA was added to both compound solution and the reference solution to eliminate the absorbance of CT-DNA itself. From the absorption titration data, the binding constant (*K*_*b*_) was determined using the equation [20]:

(1) $$\begin{array}{c} \frac{\left[\text{DNA}\right]}{\left(\varepsilon_{a}-\varepsilon_{f}\right)}=\frac{\left[\text{DNA}\right]}{\left(\varepsilon_{b}-\varepsilon_{f}\right)}\;+\frac{1}{K_{b}\left(\varepsilon_{b}-\varepsilon_{f}\right)}, \end{array}$$

where [DNA] is the concentration of CT-DNA in base pairs, *ε*_*a*_ corresponds to the extinction coefficient observed (*A*_obsd_/[*M*]), *ε*_*f*_ corresponds to the extinction coefficient of the free compound, *ε*_*b*_ is the extinction coefficient of the compound when fully bound to CT-DNA, and *K*_*b*_ is the intrinsic binding constant. The ratio of slope to intercept in the plot of [DNA]/(*ε*_*a*_ − *ε*_*f*_) versus [DNA] gave the value of *K*_*b*_. 

EB emits intense fluorescence in the presence of CT-DNA, due to its strong intercalation between the adjacent CT-DNA base pairs. It was previously reported that the enhanced fluorescence can be quenched by the addition of a second molecule [21, 22]. The extent of fluorescence quenching of EB bound to CT-DNA can be used to determine the extent of binding between the second molecule and CT-DNA. The competitive binding experiments were carried out in the buffer by keeping [DNA]/[EB] = 1.13 and varying the concentrations of the compounds. The fluorescence spectra of EB were measured using an excitation wavelength of 520 nm, and the emission range was set between 550 and 750 nm. The spectra were analyzed according to the classical Stern-Volmer equation [23]:

(2) $$\frac{I_{0}}{I}=1+K_{\text{sv}}\left[Q\right],$$

where *I*_0_ and *I* are the fluorescence intensities at 599 nm in the absence and presence of the quencher, respectively, *K*_sv_ is the linear Stern-Volmer quenching constant, and [*Q*] is the concentration of the quencher. In these experiments, [CT-DNA] = 2.5×10^−3 ^mol/L and [EB] = 2.2 × 10^−3 ^mol/L.

Viscosity experiments were conducted on an Ubbelohde viscometer, immersed in a water bath maintained at 25.0 ± 0.1°C. Titrations were performed for the compound (3 *μ*M), and each compound was introduced into CT-DNA solution (50 *μ*M) present in the viscometer. Data were presented as (*η*/*η*_0_)^1/3^ versus the ratio of the concentration of the compound to CT-DNA, where **η** is the viscosity of CT-DNA in the presence of the compound and *η*_0_ is the viscosity of CT-DNA alone. Viscosity values were calculated from the observed flow time of CT-DNA containing solutions corrected from the flow time of buffer alone (*t*_0_), *η* = (*t* − *t*_0_) [24].

#### 2.1.2. Antioxidation Studies

The hydroxyl radicals in aqueous media were generated through the Fenton-type reaction [25, 26]. The 3 mL reaction mixtures contained 1.0 mL of 40 ug/mL aqueous safranin, 1 mL of 1.0 mmol aqueous EDTA-Fe(II), 1 mL of 3% aqueous H_2_O_2_, and a series of quantitatively microadding solutions of the tested compound. The sample without the tested compound was used as the control. The reaction mixtures were incubated at 37°C for 30 min in a water-bath. Absorbance at 520 nm was measured, and the solvent effect was corrected throughout. The scavenging effect for OH^•^ was calculated from the following expression:

(3) $$\begin{array}{c} \text{Scavenging}\;\text{effect}\;\text{\%}=\frac{\left(A_{\text{sample}}-A_{r}\right)}{\left(A_{o}-A_{r}\right)}\times 100\text{\%}, \end{array}$$

where *A*_sample_ is the absorbance of the sample in the presence of the tested compound, *A*_*r*_ is the absorbance of the blank in the absence of the tested compound, and *A*_*o*_ is the absorbance in the absence of the tested compound and EDTA-Fe(II) [27].

A nonenzymatic system containing 0.5 mL 3.3 × 10^−5^ M VitB_2_, 1 mL 2.3 × 10^−4^ M NBT, 1 mL 0.05 M MET, and 2.5 mL 0.067 M phosphate buffer (Na_2_HPO_4_–NaH_2_PO_4_, pH = 7.8) was used to produce superoxide anion (O^2•−^), and the scavenging rate of O^2•−^ under the influence of 0.3–3.0 *μ*M tested compound was determined by monitoring the reduction in rate of transformation of NBT to monoformazan dye [28]. The reactions were monitored at 560 nm with a UV-Vis spectrophotometer, and the rate of absorption change was determined. The percentage inhibition of NBT reduction was calculated using the following equation [29]: percentage inhibition of NBT reduction = (1 − *k*′/*k*) × 100, where *k*′ and *k* present the slopes of the straight line of absorbance values as a function of time in the presence and absence of SOD mimic compound (SOD is superoxide dismutase), respectively. The IC_50_ values for the complexes were determined by plotting the graph of percentage inhibition of NBT reduction against the increase in the concentration of the complex. The concentration of the complex which causes 50% inhibition of NBT reduction is reported as IC_50_.

### 2.2. Synthesis of the Ligand and Complex

#### 2.2.1. 1,3-Bis(1-butylbenzimidazol-2-yl)-2-oxopropane (buobb)

The 1,3-bis(1-benzimidazol-2-yl)-2-oxopropane (5.56 g, 0.020 mol) (synthesized by the literature method [30]) was suspended in dry tetrahydrofuran (170 mL) and stirred under reflux with potassium (1.56 g). Iodobutane (4.84 g, 0.040 mol) was added; the solution was stirred for 2 h. The solvents were stripped to dryness and the resulting powder dissolved in distilled water. The soluble KI was removed by filtration. The undissolved substances were recrystallized from MeOH, and a colorless powder was deposited. Yield: 4.13 g (53%); m.p. 70–71°C. Anal. Calcd. for C_24 _H_30 _N_4 _O (Mr = 390): C 73.81; H 7.74; N 14.35%; found: C 73.83; H 7.72; N 14.37%. ^1^H NMR ([D_6_]DMSO, 400 MHz): **δ**= 7.55–7.65 (m, 4 H, Ph-H), 7.17–7.31 (m, 4 H, Ph-H), 4.87(s, 4 H, CH_2_), 4.19–4.26 (m, 4 H, CH_2_), 1.64–1.71 (m, 4 H, CH_2_), 1.18–1.29 (m, 4 H, CH_2_), 0.75–0.89 (m, 6 H, CH_3_). IR (selected data, KBr *ν*/cm^−1^): *ν* = 750 (*ν*_o–Ar_), 1060 (*ν*_C–O_), 1460 (*ν*_C=N_), 1614 (*ν*_C=C_). UV/Vis (DMF): *λ* = 280, 288 nm. Λ_*M*_ (DMF, 297 K): 0.87 S*·*cm^2^ *·*mol^−1^.

#### 2.2.2. Preparation of Cu(II) Complex

To a stirred solution of 1,3-bis(1-butylbenzimidazol-2-yl)-2-oxopropane (0.156 g, 0.40 mmol) in hot MeOH (5 mL) was added Cu(II) picrate (0.104 g, 0.20 mmol) in MeOH (5 mL). A deep brown crystalline product formed rapidly. The precipitate was filtered off, washed with MeOH and absolute Et_2_O, and dried in vacuo. The dried precipitate was dissolved in DMF resulting in a brown solution. The brown crystals suitable for X-ray diffraction studies were obtained by ether diffusion into DMF after several days at room temperature. Yield: 0.170 g (58.7%). Anal. Calcd. for C_66_H_78_Cu N_16_O_18_ (Mr = 1446.98): C, 54.78; H, 5.43; N, 15.49. Found (%): C, 54.77; H, 5.44; N, 15.50. Selected IR data (KBr *ν*/cm^−1^): 748 (*ν*_o–Ar_), 1099 (*ν*_C–O_), 1494 (*ν*_C=N_), 1631 (*ν*_C=C_). UV/Vis (DMF): *λ* = 281, 288. *Λ *_M_ (DMF, 297 K): 100.88 S*·*cm^2^ *·*mol^−1^.

#### 2.2.3. X-Ray Crystal Structure Determination

A suitable single crystals were mounted on a glass fiber, and the intensity data were collected on a Bruker Apex-II CCD (Japan) diffractometer with graphite-monochromatized Mo *K*α** radiation (*λ* = 0.71073 Å) at 296(2) K. Data reduction and cell refinement were performed using Saint programs [31]. The absorption correction was carried out by empirical methods. The structure was solved by direct methods and refined by full-matrix least squares against *F* ^2^ using Shelxtl software [32]. All H atoms were found in difference electron maps and were subsequently refined in a riding model approximation with C–H distances ranging from 0.95 to 0.99 Å. The crystal data and experimental parameters relevant to the structure determination are listed in Table 1. Selected bond distances and angles are presented in Table 2. 

## 3. Results and Discussion

### 3.1. Characterization of the Complex

The ligand buobb and the Cu(II) complex are very stable in the air. The ligand is soluble in organic solvents but insoluble in water. The Cu(II) complex is soluble in DMF and DMSO but insoluble in water and other organic solvents, such as methanol, ethanol, acetone, petroleum ether, trichloromethane, and so forth. The results of the elemental analyses show that the composition is [Cu(buobb)_2_](pic)_2_ *·*2DMF. The value of molar conductance shows that the complex is a 1 : 2 electrolyte in DMF [33]. 

The IR spectra of the free ligand and the Cu(II) complex were compared. The IR spectrum of the complex shows that the strong absorption *ν*_C=N_ in the free ligand is shifted to lower wave numbers in the Cu(II) complex. The redshift indicates that the nitrogen atoms of the ligands are coordinated to the copper (II) ions. They are the preferred nitrogen atoms for coordination, as found in other metal complexes with benzimidazole open chain crown ether derivatives [34]. This fact agrees with the result determined by X-ray diffraction. In the UV/Vis spectra, the band of free ligand is red shifted in the complex and shows clear evidence of C=N coordination to the copper atom. The absorption band is assigned to *π*-π* (imidazole) transition [35]. 

**Figure 1:**
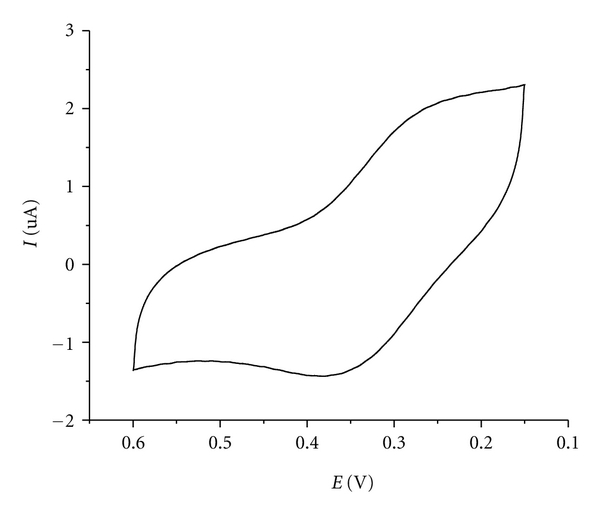
Cyclic voltammogram of the Cu(II) complex recorded with a platinum electrode in DMF solution containing (*n-*Bu)_4_N*·*ClO_4_ (0.1 M). (Scan rate = 0.05 V*·*s^−1^).

The electrochemical properties of the complex were studied by cyclic voltammetry (CV) in DMF. The voltammogram is shown in Figure 1. The Cu(II) complex exhibits a pair of cathodic and anodic waves. The separation between the cathodic and anodic peak potentials Δ*E*_p_  (Δ*E*_pa_ − *E*_pc_) and the current *I *(*i*_pa_/*i*_pc_) indicates a semi*­*reversible redox process assignable to the Cu^II^/Cu^I^ couple [36]. The neutral uncomplexed ligand buobb is proved not to be electroactive over the range −1.3 to +1.3 V. According to previous reports [37], a transition metal complex must have a reduction potential below 0.65 V [*E*°  (^1^O_2_–O_2_^−^)] and above −0.33 V [*E*°  (O_2_–O_2_^−^)] such that catalysis can take place to be an effective mimic of superoxide dismutase but toxic single oxygen cannot be formed; so the redox potential 0.139 V shows that the complex has SOD activity.

### 3.2. Description of the Structure of the Complex

**Figure 2:**
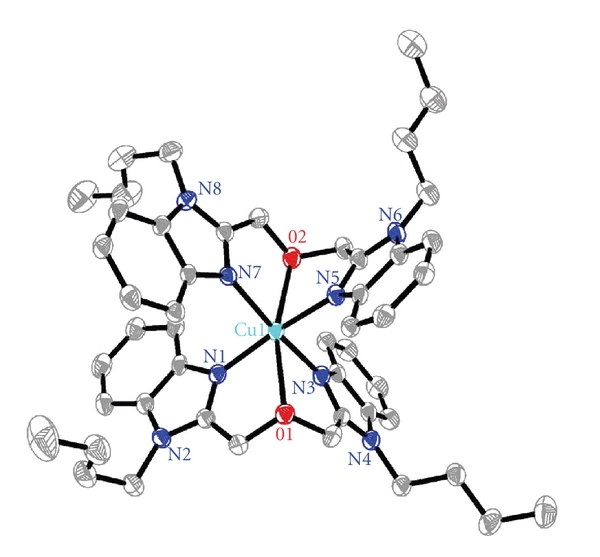
Molecular structure of the [Cu(buobb)_2_]^2+^ cation with displacement ellipsoids drawn at the 30% probability level; H atoms, trinitrophenol anions and DMF are omitted for clarity.

**Figure 3:**
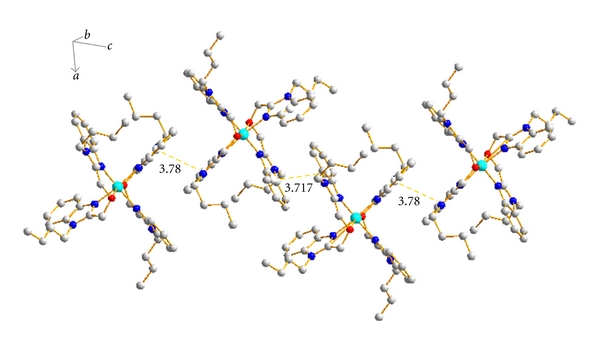
The structure of complex linked via *π*-*π* stacking interaction.

Complex crystallizes in the Triclinic space group P-1, and its structure along with the atomic numbering scheme is shown in Figure 2, consisting of a [Cu(buobb)_2_]^2+^ cation, two trinitrophenol anions, and two DMF. The central metal ion of [Cu(buobb)_2_]^2+^ cation, adopting a distorted octahedral geometry, is six coordinated with an N_4_O_2_ ligand set in which four N atoms (N(1), N(3), N(5), and N(7)) are afforded by the benzimidazole rings and other two O atoms (O(1) and O(2)) are supplied by the buobb. An equatorial plane is formed by atoms N1, N3, N5, N7, and Cu1, where the largest deviation for N7 atom is 0.233 Å and the deviation for the Cu(1) atom is 0.021 Å from the mean plane. The bond angles of ideal 90° are range from 89.82(15) [N(1)–Cu(1)–N(3)] to 90.69(15) N(1)–Cu(1)–N(7) and from 92.49(14) N(3)–Cu(1)–O(2) to 75.17(14) N(3)–Cu(1)–O(1). With regard to a regular octahedron, the angles show a certain distortion. The benzimidazole rings from the adjacent units arrange in an offset face-to-face fashion with the vertical distance of center to center 3.780 Å and 3.717 Å, suggesting significant **π**-**π**stacking interactions (Figure 3). Thus, the units are stacked together efficiently via the **π**-**π** stacking interactions of benzimidazole.

### 3.3. DNA-Binding Properties

#### 3.3.1. Absorption Spectroscopic Studies

**Figure 4:**
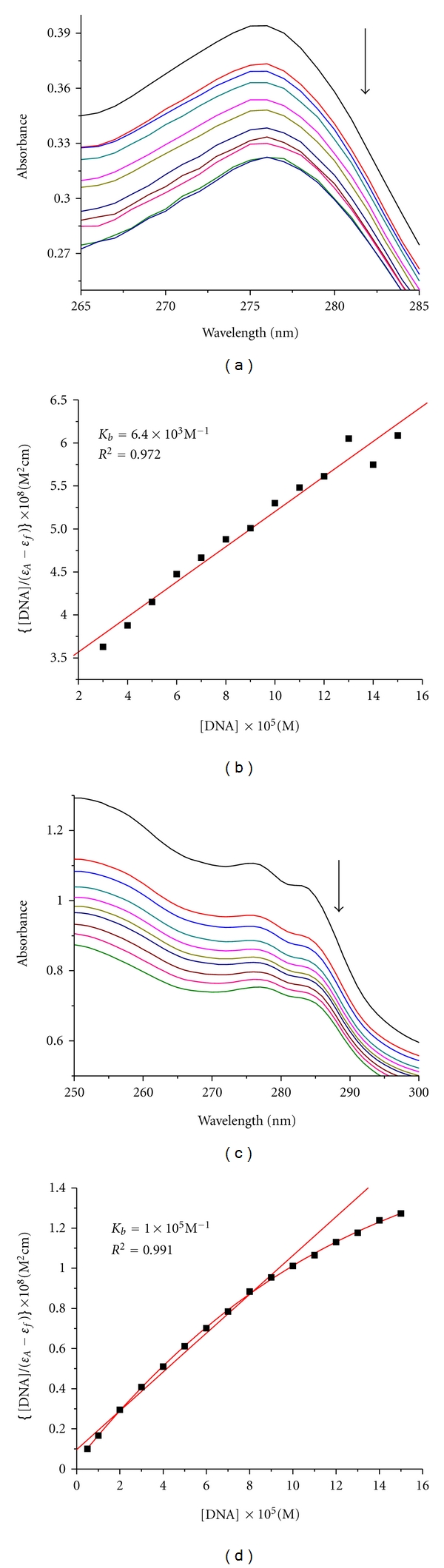
Electronic spectra of the free ligand buobb (a) and the complex Cu(II) (c) in Tris-HCl buffer upon addition of CT-DNA. The arrow shows the emission intensity changes upon increasing DNA concentration. [DNA]/(*ε*_*a*_ − *ε*_*f*_) versus [DNA] for the titration of the ligand buobb (b) and the complex Cu(II) (d) with CT-DNA.

The electronic absorption spectra of the ligand and the Cu(II) complex in the absence and presence of CT-DNA are given in Figure 4. As seen from Figure 4(a), there exists a band at 277 nm for the free ligand, and in Figure 4(c) a band is at 278 nm for the Cu(II) complex. With increasing concentrations of CT-DNA, the absorption bands of the free ligand at 277 nm exhibited hypochromism of 18.3%. For the Cu(II) complex, the absorption band at 278 nm appeared with hypochromism of 32.1%. The hypochromisms observed for the bands of the free ligand and its Cu(II) complex are accompanied. The electronic absorption spectroscopy is one of the most useful techniques in DNA binding studies of metal complexes [38–40]. A compound binding to DNA through intercalation usually results in hypochromism due to the intercalation mode involving a strong *π*-*π* stacking interaction between the aromatic chromophore and the DNA base pairs. It seems to be generally accepted that the extent of the hypochromism in the UV-vis band is consistent with the strength of intercalative interaction [41]. So, the above phenomena imply that the compounds interact with CT-DNA quite probably by intercalating into the DNA base pairs. From the results of electronic absorption spectroscopy, the *K*_*b*_ values of the free ligand buobb and the Cu(II) complex were 6.4 × 10^3^ M^−1^ (*R* ^2^ = 0.972 for 13 points) and 1.0 × 10^5^ M^−1^ (*R* ^2^ = 0.991 for 10 points), respectively. We can conclude that the free ligand buobb and the Cu(II) complex can interact with CT-DNA through the same mode (intercalation). In general, this may be attributed to the Cu(II) complex with the electric effect and steric hindrance resulting in the relatively close stacking between the Cu(II) complex and the DNA base pairs [42, 43].

#### 3.3.2. Fluorescence Spectroscopic Studies

**Figure 5:**
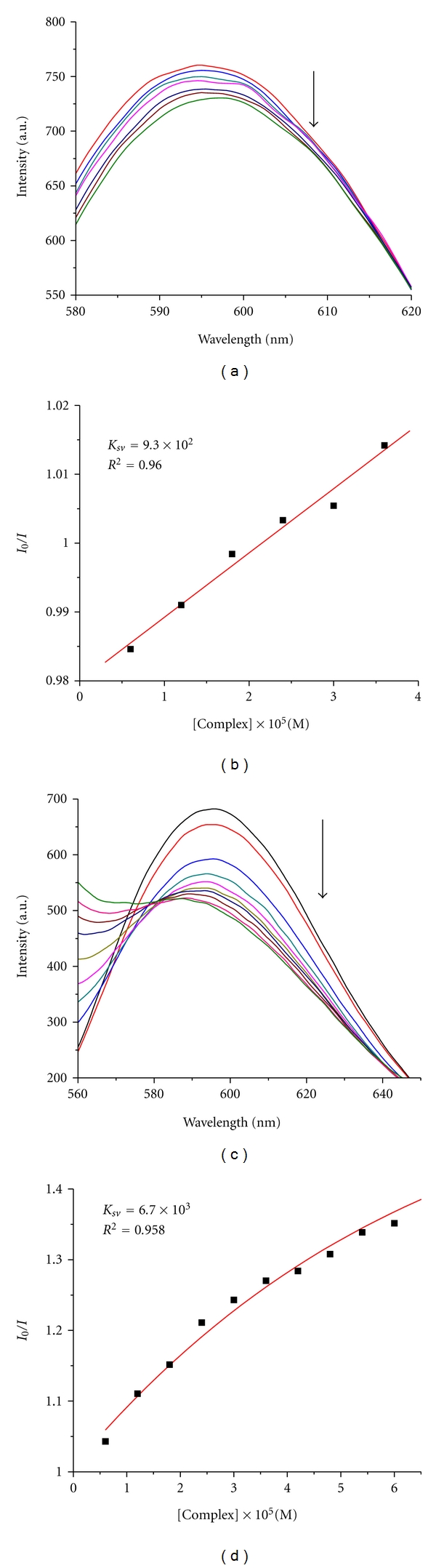
Emission spectra of EB bound to CT-DNA in the presence of the free buobb (a) and the Cu(II) complex (c), *λ*_ex_ = 520 nm. The arrows show the intensity changes upon increasing concentrations of the complexes. Fluorescence quenching curves of EB bound to CT-DNA by the free buobb (b) and the Cu(II) complex (d). (Plots of *I*_0_/*I* versus [Complex]).

In order to examine the ability of the compounds to displace EB (=3,8-Diamino-5-ethyl-6-phenyl-phenanthridinium bromide) from its EB-DNA complex, a competitive EB binding study has been undertaken with UV-vis and fluorescence experiments [44, 45]. EB, a phenanthridine fluorescence dye, is a typical indicator of intercalation [46] that forms soluble complexes with nucleic acids and emits intense fluorescence in the presence of CT DNA due to the intercalation of the planar phenanthridine ring between adjacent base pairs on the double helix. The changes observed in the spectra of EB on its binding to CT DNA are often used for the interaction study between DNA and other compounds, such as metal complexes [47]. The ligand buobb and the Cu(II) complex show no fluorescence at room temperature in solution or in the presence of CT-DNA, and their binding to DNA cannot be directly predicted through the emission spectra. However, competitive EB binding studies could be undertaken in order to examine the binding of each compound with DNA. EB does not show any appreciable emission in buffer solution due to fluorescence quenching of the free EB by the solvent molecules. Upon addition of the ligand or complex to a solution containing EB, neither quenching of free EB fluorescence has been observed, nor new peak in the spectra appeared. The fluorescence intensity is highly enhanced upon addition of CT-DNA, due to its strong intercalation with DNA base pairs. Addition of a second molecule, which may bind to DNA more strongly than EB, results in a decrease in the DNA-induced EB emission due to the replacement of EB or to electron transfer [48]. The addition of the compounds results in a significant decrease of the fluorescence intensity of the emission band of the DNA-EB system at 599 nm indicating the competition of the compounds with EB in binding to DNA. The observed quenching of DNA-EB fluorescence for the ligand or their complex suggests that they displace EB from the DNA-EB complex and they can interact with CT DNA probably by the intercalative mode. The Stern-Volmer plots of DNA-EB (Figure 5) illustrate that the quenching of EB bound to DNA by the compounds with the linear Stern-Volmer equation, which proves that the replacement of EB bound to DNA by each compound results in a decrease in the fluorescence intensity. The *K*_sv_ values of the ligand buobb and the Cu(II) complex show that they can displace EB and bind to the DNA [44, 45]. The *K*_sv_ values for the ligand buobb and the Cu(II) complex are 9.2 × 10^2^ M^−1^ (*R* ^2^ = 0.960 for 6 points) and 6.7 × 10^3^ M^−1^ (*R* ^2^ = 0.958 for 6 points), respectively. The data suggest the interaction. Moreover, the binding strength of Cu(II) complex is greater than the free ligand.

#### 3.3.3. Viscosity Measurements

**Figure 6:**
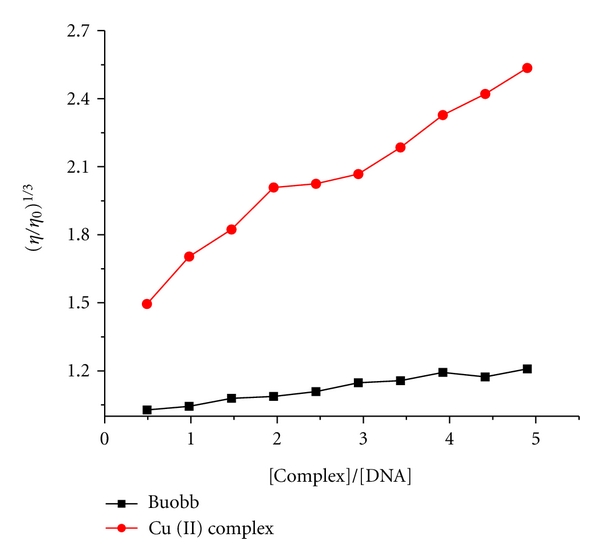
Effect of increasing amounts of the compounds on the relative viscosity at 25.0 ± 0.1°C.

Optical photophysical techniques are widely used to study the binding model of the ligand, metal complexes, and DNA, but do not give sufficient clues to support a binding model. Therefore, viscosity measurements were carried out to further clarify the interaction of metal complexes, and DNA. Hydrodynamic measurements that are sensitive to the length change (i.e., viscosity and sedimentation) are regarded as the least ambiguous and the most critical tests of a binding model in solution in the absence of crystallographic structural data [49, 50]. For the ligand buobb and the Cu(II) complex, with increasing the amounts of compounds, the viscosity of DNA increases steadily. The values of (*η*/*η*_0_)^1/3^ were plotted against [compound]/[DNA] (Figure 6). In classical intercalation, the DNA helix lengthens as base pairs are separated to accommodate the bound ligand leading to increased DNA viscosity whereas a partial, nonclassical ligand intercalation causes a bend (or kink) in DNA helix reducing its effective length and thereby its viscosity [49]. The effects of the ligand and the Cu(II) complex on the viscosity of CT-DNA are shown in Figure 6. The viscosity of CT-DNA is increased steadily with the increment of the ligand buobb and the Cu(II) complex, and it is further illustrated that the ligand buobb and the Cu(II) complex intercalate with CT-DNA [49]. The results from the viscosity experiments confirm the mode of these compounds intercalating into DNA base pairs and already established through absorption spectroscopic studies and fluorescence spectroscopic studies.

### 3.4. Antioxidant Activity

#### 3.4.1. Hydroxyl Radical Scavenging Activity

**Figure 7:**
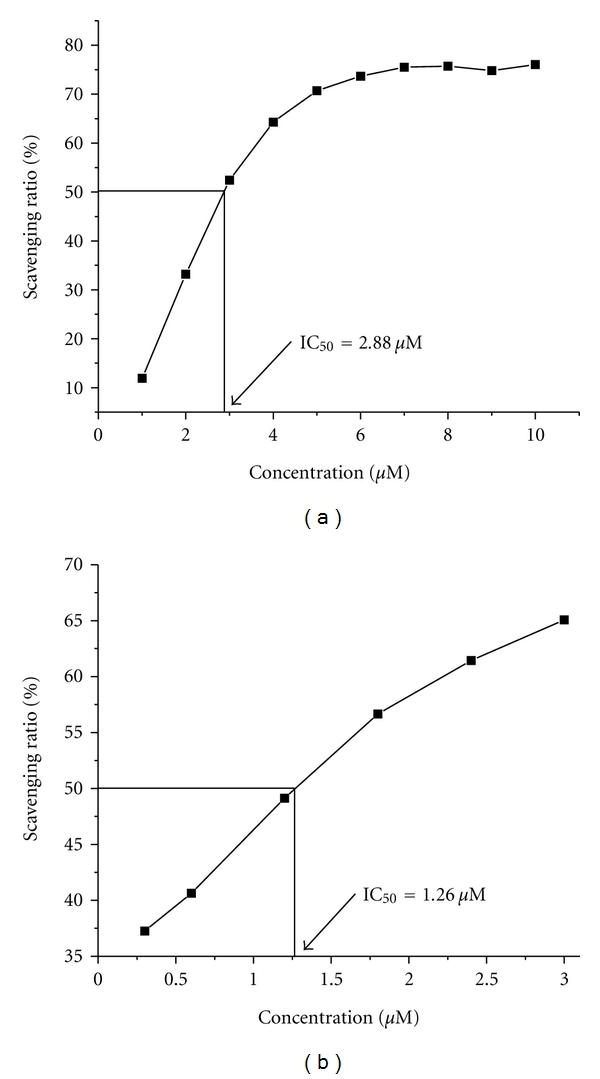
Plots of antioxidation properties for the Cu(II) complex. (a) Represents the hydroxyl radical scavenging effect (%) for the Cu(II) complex. (b) Represents the superoxide radical scavenging effect (%) for the Cu(II) complex.

Figure 7(a) depicts the inhibitory effect of the Cu(II) complex on OH^•^ radicals. The inhibitory activity of the Cu(II) complex is marked, and the suppression ratio increases with increasing concentration of the test Cu(II) complex. We compared the present complex with the well-known natural antioxidants mannitol and vitamin C, using the same method as reported in a previous paper [50]. The 50% inhibitory concentration (IC_50_) value of mannitol is about 9.6 *μ*M. According to the antioxidant experiments, the IC_50_ of the Cu(II) complex is 2.88 *μ*M (Figure 7(a)), which implies that the Cu(II) complex exhibits the ability to scavenge hydroxyl radical as well as mannitol and vitamin C. This may be attributed to the Cu(II) ion's valence alternation [51, 52].

#### 3.4.2. Superoxide Radical Scavenging Activity

We have examined the SOD activity of complex using the xanthine/xanthine oxidase assay due to their conducive solubility. SOD activity was monitored by reduction of nitro blue tetrazolium (NBT) with O_2_^−•^ generated by xanthine/xanthine oxidase system. As the reaction proceeds, the farmazan color is developed and a color change from colourless to blue appeared which was associated with an increase in the absorbance at 560 nm.  Figure 7(b) shows the plot of superoxide radical scavenging effects (%) for the Cu(II) complex. The ligand does not have antioxidant activity; the values of IC_50_ of the Cu(II) complex for O_2_^−•^ are 1.26 *μ*M. These results suggest that complex shows stronger scavenging effects than the free ligand buobb for O_2_^−•^. We suggest that the mechanism of action of the Cu(II) complex involves the redox process. The IC_50_ values for [Cu(salicylate)_2_], [Cu(NA/Sal)] (NA/Sal = nicotinic-salicylic acid), CuSO_4_, and the native Cu, Zn-SOD [53, 54] are 16 *μ*M, 42.79 *μ*M, 30 *μ*M, and 0.04 *μ*M, respectively. The IC_50_ values for the copper complex was better than the values reported for [Cu(salicylate)_2_], [Cu(NA/Sal)], and CuSO_4_, less than native Cu, Zn-SOD. The results and the electrochemical properties are consistent.

## 4. Conclusion

Taken together, a new bis-benzimidazole base ligand and its Cu(II) complex were reported. The structure of the ligand and the Cu(II) complex were determined on the basis of elemental analyses, molar conductivities, IR spectra, ^1^H NMR, and UV-vis spectra. Cu(II) complex's crystal structure has been determined by X-ray crystallography method. Experimental results indicate that the ligand and the Cu(II) complex bind to DNA via an intercalation mode and the Cu(II) complex can bind to DNA more strongly than the free ligand alone. Moreover, the Cu(II) complex has better antioxidant activity than the ligand. Results obtained from our present work would be useful to understand the mechanism of interactions of the small molecule compounds binding to DNA and helpful in the development of their potential biological, pharmaceutical, and physiological implications in the future.

##  Supporting Information Available

Crystallographic data (excluding structure factors) for the structure in this paper have been deposited with the Cambridge Crystallographic Data Centre as supplementary publication CCDC 835138. Copies of the data can be obtained, free of charge, on application to the CCDC, 12 Union Road, Cambridge CB2 1EZ, UK. Tel: +44-01223-762910; fax: +44-01223-336033; e-mail: deposit@ccdc.cam.ac.uk or http://www.ccdc.cam.ac.uk.

## Figures and Tables

**Table 1:** Crystallographic data for [Cu(buobb)_2_](pic)_2_ *·*2DMF.

Compound	[Cu (buobb_2_)_2_](pic)_2_ *·*2DMF
Molecular formula	C_66_H_78_CuN_16_O_18_
Molecular weight	1446.98
Crystal system	Triclinic
Space group	P-1
*a* (Å)	14.0047 (14)
*b* (Å)	16.1337 (17)
*c* (Å)	16.6316 (17)
*α* (°)	70.8150 (10)
*β* (°)	78.0420 (10)
*γ* (°)	74.7260 (10)
*V *(Å^3^)	3393.7 (6)
*Z*	2
*ρ*_cald_ (mg*·*m^−3^) Absorption coefficient (mm^−1^)	1.416 0.406
*F* (000)	1518
Crystal size (mm)	0.35 × 0.31 × 0.29
**θ** range for data collection (°)	1.52 to 25.00
*h/k/l* (max, min)	−16 ≤ *h* ≤ 16, − 19 ≤ *k* ≤ 19, − 19 ≤ *l* ≤ 19
Reflections collected	24488
Independent reflections	11850
Refinement method	Full-matrix least squares on *F* ^2^
Data/restraints/parameters	11850/14/918
Goodness of fit on* F * ^2^	1.044
Final *R*_1_, *wR*_2_ indices [*I* > 2*σ*(*I*)]	*R*_1_ = 0.0837, *wR*_2_ = 0.2320
*R*_1_, *wR*_2_ indices (all data)	*R*_1_ = 0.1184, *wR*_2_ = 0.2701
Largest differences peak and hole (eÅ^−3^)	1.107 and −0.658

**Table 2:** Selected bond lengths (Å) and angles (°) for [Cu(buobb)_2_](pic)_2_ *·*2DMF.

Bond lengths	Cu(1)–N(1)	1.973(4)	Cu(1)–N(3)	2.032(4)
	Cu(1)–N(5)	1.998(4)	Cu(1)–N(7)	1.993(4)
	Cu(1)–O(1)	2.486(3)	Cu(1)–O(2)	2.492(4)
Bond angles	N(1)–Cu(1)–N(7)	90.69(15)	N(1)–Cu(1)–N(5)	167.90(17)
	N(7)–Cu(1)–N(5)	89.86(15)	N(1)–Cu(1)–N(3)	89.82(15)
	N(7)–Cu(1)–N(3)	165.70(17)	N(5)–Cu(1)–N(3)	92.62(15)
	N(1)–Cu(1)–O(1)	74.27(14)	N(7)–Cu(1)–O(1)	118.65(14)
	N(5)–Cu(1)–O(1)	94.90(14)	N(3)–Cu(1)–O(1)	75.17(14)
	N(1)–Cu(1)–O(2)	116.15(15)	N(7)–Cu(1)–O(2)	74.50(14)
	N(5)–Cu(1)–O(2)	75.59(14)	N(3)–Cu(1)–O(2)	92.49(14)
	O(1)–Cu(1)–O(2)	164.24(11)		
